# The Role of Screening, Risk Factors, and Early Intervention in Preventing Diabetes in the Obese Population: A Systematic Review

**DOI:** 10.7759/cureus.63952

**Published:** 2024-07-06

**Authors:** Noor A Merwass, Yazed K Alkhader, Salma A Alharthi, Rawdha M Al Fardan, Abdullah M Alqahtani, Fahad A Mahnashi, Nora M Salam, Mustafa M Al Najim, Ahmad A Alenezi, Abdullah O Binobaid

**Affiliations:** 1 Department of Internal Medicine, Al Thager Hospital, Jeddah, SAU; 2 Family Medicine, Riyadh Second Health Cluster, King Fahad Medical City, Riyadh, SAU; 3 Department of Internal Medicine, King Abdulaziz Specialist Hospital, Taif, SAU; 4 Department of Internal Medicine, Salmaniya Medical Complex, Manama, BHR; 5 College of Medicine, Imam Abdulrahman Bin Faisal University, Dammam, SAU; 6 Department of Family Medicine, Medical Administration at Presidency of State Security, Riyadh, SAU; 7 Nursing Department, King Salman Hospital, Riyadh, SAU; 8 Department of General Medicine, Locumlimk Healthcare Agency, Mullingar, IRL; 9 Department of Primary Care, Al-Jahra Health Region, Al Jahra, KWT; 10 Department of Internal Medicine, Security Forces Hospital, Riyadh, SAU

**Keywords:** diabetes type 2, intervention, prevention, risk factors, obesity, diabetes

## Abstract

With its rising global prevalence, diabetes has become one of the most significant and challenging health problems afflicting the world's population today. The increasing burden of diabetes and its associated complications calls for immediate action for prevention which primarily includes addressing the risk factors. The most significant risk factor for the onset of diabetes is obesity. Obesity and diabetes rates have been rising simultaneously, posing a threat to patient mortality and driving up community healthcare costs. A weight loss of five percent or more of total body weight has been shown to improve the quality of life, reduce the need for pharmacological therapy for diabetes, and enhance glycemic control. This level of weight loss can have significant health benefits, particularly for individuals with diabetes or at risk for developing diabetes. We aim to conduct this systematic review to assess diverse risk factors contributing to the incidence of diabetes among the obese population and determine various preventive strategies and recommendations in practice for the prevention of diabetes in this cohort. As a result, we included original studies that recruited the obese and diabetic populations and defined preventive measures for early intervention. Additionally, we included studies published in the last 10 years (2014-2024) only for the latest evidence. Studies including obese populations with cardiovascular diseases and neurological disorders were excluded. The Newcastle-Ottoman Castle assessment tool was utilized to assess the quality of the studies. We included nine studies that recruited 60,645 patients and were published between 2015 and 2022. Findings suggest that obesity alone is a significant contributor to the occurrence or onset of diabetes. At the same time, the presence of other risk factors, including hypertension, dyslipidemia, elevated triglycerides, or HDL and LDL levels, may further increase the risk of diabetes and its associated complications among the obese population. Preventive strategies emphasize early intervention through increasing awareness and educating communities about risk factors and lifestyle interventions, including the limitations of fast food diets for the prevention of diabetes and weight control. Since obesity is considered to be an independent risk factor for the development of diabetes, addressing and managing it is of critical importance clinically. Targeted early interventions, including screening for risk factors, health promotion, and education activities, can aid in the adaptation of healthier lifestyles, which can reduce the burden of these diseases significantly.

## Introduction and background

Diabetes mellitus is a chronic, multifaceted metabolic disease characterized by hyperglycemia or elevated blood glucose levels resulting from abnormalities in insulin secretion, insulin action, or both. Diabetes presents in diverse ways and further leads to metabolic dysfunctions of proteins, fats, and carbohydrates. The primary contributory factor to diabetes-related morbidity and death is long-term hyperglycemia, which frequently results in a myriad of microvascular and macrovascular diabetic complications. With its rising global prevalence, diabetes has become one of the most significant and challenging health problems afflicting the world's population today. Nearly everywhere in the world, the prevalence of diabetes has increased in tandem with economic growth, which has fueled urbanization and the uptake of contemporary lifestyles [1]. Diabetes is primarily classified into four types: Type 1 diabetes results from an absolute absence of insulin secretion; Type 2 diabetes, which is significantly more prevalent, is caused by a combination of insufficient insulin secretory response and resistance to insulin action; and Type 1.5 diabetes, also known as latent autoimmune diabetes in adults (LADA), presents characteristics of both Type 1 and Type 2 diabetes, typically developing in adulthood with a slower progression to insulin dependency. Additionally, gestational diabetes is recognized as a form of diabetes that refers to any degree of glucose intolerance that initially manifests during pregnancy. Most instances of gestational diabetes resolve after delivery, but the criteria for diagnosis apply regardless of whether the condition continues postpartum and do not rule out the potential for undiagnosed glucose intolerance before or coincident with the pregnancy [2].

Following estimates for global diabetes prevalence, almost 10.5% of all individuals in the world (536.6 million) aged 20 to 79 had diabetes in 2021; by 2045, this percentage is projected to increase to 12.2% (783.2 million). The prevalence of diabetes was highest in people 75-79 years old, and it was comparable in men and women [3]. Moreover, based on data from the World Health Organization, Saudi Arabia has the second-highest diabetes rate in the Middle East and the seventh highest worldwide. Approximately 3 million people have pre-diabetes, while 7 million people are expected to have diabetes. Perhaps even more concerning is the rising diabetes trend that has recently been seen in Saudi Arabia. In Saudi Arabia, the prevalence of diabetes has increased by about ten times over the last three eras. Diabetes mellitus has been linked to poor overall health, worse quality of life, and high rates of death, morbidity, and vascular complications. Diabetes is rapidly growing to alarming levels in Saudi Arabia and is now a major contributor to complications and fatalities [4]. 

Type 2 diabetes is frequently associated with cardiovascular disease, which can be fatal, especially in cases of coronary artery disease, stroke, and heart failure, and needs to be regarded as a separate cardiovascular risk factor. Nephropathy has a diverse etiology but is prevalent in type 2 diabetes. It is currently the main factor leading to end-stage kidney disease. Additionally, retinopathy, which paradoxically progresses slowly, must be detected and treated in elderly, high-risk ophthalmology patients. A serious repercussion of microangiopathy and neuropathy is diabetic foot. It could be viewed as a super complication comprised of several complications. Furthermore, several emerging complications, including mood disorders, sleep apnea syndrome, cognitive impairment, and bone metabolism impairments, are also observed among diabetic patients [5]. A complicated combination of genetic predisposition, environmental conditions, and other risk factors like obesity and a sedentary lifestyle leads to the incidence of type 2 diabetes. Numerous diabetes risk factors, life-threatening complications, delayed diagnosis until micro- and macrovascular complications arise, current therapy failures, and high treatment costs necessitate the development of new, effective treatment strategies and appropriate preventive measures in order to control and manage diabetes [6].

As obesity and diabetes rates rise concurrently, they pose a significant threat to patient mortality and drive up community healthcare costs. Studies indicate that losing 5% or more of total body weight can improve quality of life, reduce the need for pharmacological therapy for diabetes, and enhance glycemic control [7]. Individuals with a body mass index (BMI) of 30 kg/m^2^ or greater are considered obese. Over the past three decades, the global obesity prevalence has risen by 47.1% for children and 27.5% for adults. Obesity rates are influenced by lifestyle choices, urbanisation, and consumption trends [8]. The pathophysiologic basis of the relationship between diabetes mellitus and obesity is complex and multifactorial, involving a combination of genetic, environmental, and metabolic factors. Adiposity-induced changes in β cell function, adipose tissue biology, and multi-organ insulin resistance are among the complex cellular and physiological mechanisms that underlie the relationship between obesity and type 2 diabetes. These mechanisms can often be improved and even return to normal with sufficient weight loss [9].

Research studies have demonstrated that individuals who are more susceptible to type 2 diabetes can prevent the progression of the disease. Diabetes has been shown to develop less frequently in diverse at-risk populations when lifestyle modification interventions are implemented, including a 5%-10% reduction in excess body weight and an increase in moderate physical activity of 150 minutes per week [10]. This signifies the importance of addressing the risk of diabetes in the obese population since it is crucial for prevention as it helps improve insulin sensitivity, enhance metabolic and cardiovascular health, promote healthy aging, reduce healthcare costs, and improve quality of life. This underscores the importance of comprehensive strategies aimed at addressing obesity as a key component of diabetes prevention efforts. Therefore, through this study, we aim to systematically review the existing literature in terms of the uptake of various preventive measures, including screening, assessment of risk factors, and early intervention targeted at reducing the risk of diabetes among obese populations. Additionally, we aim to provide deep insights into the mechanisms behind the association between diabetes and obesity. The findings can aid in the development of efficacious and targeted preventive strategies that can further help in reducing the burden of these two significant diseases.

## Review

Methods

Definition of Outcomes, and Inclusion and Exclusion Criteria

Our study aimed to assess various risk factors contributing to the incidence of diabetes among the obese population and to determine preventive strategies and recommendations for diabetes prevention in this cohort. Consequently, we included original studies that met the following criteria: recruited participants who were obese (defined as a Body Mass Index (BMI) ≥ 30) and had diabetes or were at risk of developing diabetes; evaluated specific preventive measures for diabetes, such as lifestyle interventions (e.g., diet and exercise), pharmacological treatments, or early screening programs; provided outcomes related to diabetes prevention, such as reduction in diabetes incidence, improvement in glycemic control, or reduction in diabetes-related complications; were published within the last 10 years (2014-2024) to ensure the inclusion of recent evidence; and were written in English or had an available English translation. We excluded studies if they focused on obese populations with primary diagnoses of cardiovascular diseases or neurological disorders, as these conditions could confound the specific relationship between obesity and diabetes. Additionally, we excluded case reports with small sample sizes (less than 10 participants) or those lacking descriptive statistics, as well as nonhuman or laboratory studies, non-original or incomplete investigations, abstract-only articles, protocols, theses, or reviews.

Search Strategy

To identify relevant studies, we conducted a comprehensive manual screening to extract relevant keywords for crafting an optimal search query. Our search strategy included terms such as (diabetes mellitus OR diabetes OR diabetics) AND (obesity OR obese OR increased body mass index OR increased BMI) AND (risk OR risk factors) AND (prevention OR prevent OR screening) AND (early intervention OR early management OR early treatment) AND (recommendation OR strategy OR guidelines). We utilized the following databases for our search: PubMed, Science Direct, Web of Science, and Cochrane Library. We limited our search to the titles and abstracts of retrieved results to maximize the relevance of the studies. We used EndNote software (Clarivate, St. Helier, Jersey) to compile and eliminate duplicates across all databases. Additionally, we manually scrutinized the reference lists of included studies, related reviews, and relevant sections of comparable articles in PubMed to identify any potentially overlooked papers. Our systematic review followed the Preferred Reporting Items for Systematic Reviews and Meta-Analyses (PRISMA) guidelines.

Screening and Extraction

Articles with irrelevant titles were excluded from consideration. In the subsequent phase, both the full text and abstracts of the papers were meticulously reviewed to determine their compliance with the inclusion criteria. Titles and abstracts were organized, assessed, and scrutinized for duplicate entries using EndNote X8 reference management software. We adopted a dual screening approach: one screening for the evaluation of titles and abstracts, and another for the comprehensive examination of the entire texts. Once all relevant articles were identified, a structured extraction sheet was created to capture pertinent information aligned with our specific objectives. This included study design (e.g., randomized controlled trial, cohort study), study period and duration of follow-up, risk factors assessed (e.g., dietary habits, physical activity, genetic predispositions), participant characteristics (e.g., age, gender, BMI), number of participants with defined obesity and diabetes, geographical location of the study, and outcomes measured (e.g., incidence of diabetes, glycemic control, complication rates).

Quality Assessment

We collected data from the encompassed studies to evaluate the potential bias they might entail. For assessing the quality of observational studies, we employed the modified Newcastle-Ottawa Scale (NOS), which evaluates four domains: selection of study groups, comparability of the groups, and the ascertainment of either the exposure or outcome of interest. Each domain encompasses specific criteria: Selection involves assessing the representativeness of the exposed cohort, the selection of the non-exposed cohort, the ascertainment of exposure, and the demonstration that the outcome of interest was not present at the start of the study. Comparability evaluates the comparability of cohorts based on the design or analysis, controlling for confounding factors. Outcome/Exposure Assessment examines the assessment of the outcome, the follow-up duration, and the adequacy of follow-up. Reporting focuses on the clarity and thoroughness of reporting the results. Each study was rated on a scale from 0 to 10, with higher scores indicating a lower risk of bias. The scores were then categorized as excellent (9-10), good (7-8), satisfactory (5-6), or unsatisfactory (0-4).

To ensure consistency and reliability in the quality assessment, two independent reviewers evaluated each study using the NOS. Any discrepancies between the reviewers were resolved through discussion or by involving a third reviewer. This rigorous approach ensured that all relevant aspects of study quality were thoroughly evaluated and that potential biases were systematically identified and addressed.

Results

Search Results

**Figure 1:**
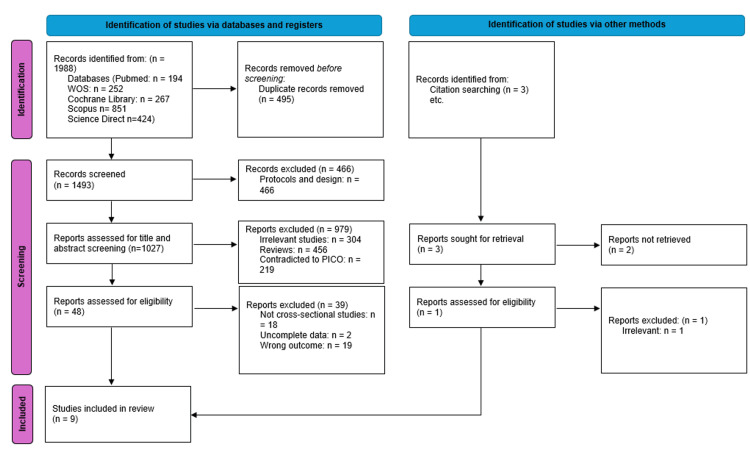
PRISMA flowchart PRISMA: Preferred Reporting Items for Systematic Reviews and Meta-Analyses

We executed the search methodologies outlined previously, resulting in the identification of a total of 1,988 citations, which were subsequently reduced to 1,493 following the removal of duplicates. Upon screening titles and abstracts, only 48 citations met the eligibility criteria for further consideration. Through full-text screening, this number was further refined to nine articles aligning with our inclusion and exclusion criteria. Figure 1 provides an in-depth depiction of the search strategy and screening process.

Results of Quality Assessment

Upon evaluating the quality of the included studies, it was determined that overall, the studies exhibited good quality with minimal risk of bias. The detailed findings of the quality assessment are presented in Table 1.

**Table 1:** Summary of the results of bias assessment of the included studies using the modified Newcastle-Ottawa scale (NOS) for cross-sectional studies A study can be awarded a maximum of one star for each items within the selection and outcome categories. A maximum of two stars can be given for comparability.

Author	Year		Selection			Comparability	Outcome		Total score	Quality
		Representativeness of the Sample	Sample size	Non- Respondents	Ascertainment of the Exposure	The Subjects in Different Outcome Groups are Comparable	Assessment of outcome	Statistical analysis		
Chowdhury et al. [11]	2022	+	+		+	++	+	+	7	Good
Shrestha et al. [12]	2022	+	+	+	+	+	+	+	7	Good
Al-Thani et al. [13]	2021	+	+		+	+	+	+	6	Satisfactory
Lu et al. [14]	2021	+	+	+	+	++	+	+	8	Good
Ton et al. [15]	2020	+	+	+	+	+	+	+	7	Good
Al Mansour MA et al. [16]	2020	+	+	+	+	++	+	+	8	Good
Aldossari et al. [17]	2018	+	+	+	+	++	+	+	8	Good
Ranabhat et al. [18]	2016	+	+		+	+	+	+	6	Satisfactory
Bodicoat et al. [19]	2015	+	+	+	+	+	+	+	7	Good

Characteristics of the Included Studies

We incorporated a total of nine studies encompassing 60,645 patients, published between 2015 and 2022 [11-19]. All participants in these studies were aged 18 or older, with 53.35% male and 46.65% female. Notably, all the studies were cross-sectional. Geographically, two studies originated from Saudi Arabia and Nepal, followed by one study each from Bangladesh, Qatar, China, Vietnam, and the United Kingdom. A comprehensive summary of the baseline characteristics of these studies can be found in Table 2. Discrepancies in sample sizes across the included papers likely stem from differences in study objectives and inclusion criteria.

**Table 2:** Baseline characteristics of the included studies NR: Not reported, DRU:  Diabetes Research Unit, NIHR CLAHRC–EM =National Institute for Health Research Collaboration for Leadership in Applied Health Research and Care–East Midlands, SA: Saudi Arabia

Author	Country	Year	Study design	Study period	Total participants	Mean age	Gender(Male/Female)	Funding
Chowdhury et al. [11]	Bangladesh	2022	Cross sectional	2011 to 2018	14,376, Diabetes=1,802, Obese=3,469	≥35 years	48.98%/51.02%	NR
Shrestha et al. [12]	Nepal	2022	Cross sectional	2016 to 2018	13,200, Diabetes=884, Obese=3,866	20 years and above	42.2%/57.9%	NR
Al-Thani et al. [13]	Qatar	2021	Cross sectional	2012	2,497, DM=1,670, obese=1,086, central obesity=1,672	18–64 years	49.7%/50.3%	Qatar National Research Fund
Lu et al. [14]	China	2021	Cross sectional	2019	5,860	<60 years or ≥60 years	58.62%/41.38%	No Funding
Ton et al. [15]	Vietnam	2020	Cross sectional	2011-2017	12,725, Abdominal obesity=5,807, Obesity =3,021, Diabetes=1,522, Prediabetes=4,559	45-69 years	28.15%/71.9%	“International Cooperation & Education Program (#NCCRI· NCCI 52210-52211, 2019-2020)” of the National Cancer Center, South Korea.
Mansour et al. [16]	Saudi Arabia	2019	Cross sectional	NR	353, DM=122, Obese=300	<40 years or ≥40 years	46.5%/53.5%	No Funding
Aldossari et al. [17]	Saudi Arabia	2018	Cross sectional	2016	1,019, Diabetes=381, Obese=138	31.42 ± 9.4	100%	DRU, College of Medicine, Prince Sattam Bin Abdulaziz University, Al-Kharj, SA
Ranabhat et al. [18]	Nepal	2017	Cross sectional	2013	154	NR	NR	NR
Bodicoat et al. [19]	United Kingdom	2015	Cross sectional	2004 and 2011	10,461, ADDITION-Leicester =6,200, Prevent Diabetes =3,431, Walking Away from Diabetes = 830	59.03± 10·37	53%/47·2%	NIHR CLAHRC – EM

Study Outcome Measures

Among the nine eligible studies, several risk factors for diabetes within the study populations were thoroughly evaluated (Table 3). Notably, the highest prevalence of obesity was reported by Al Mansour MA, at a striking 42.30%, closely followed by Al-Thani et al. [13], who reported a prevalence of 41.50% . In comparison, other studies showed lower obesity prevalence rates, with frequencies of 27.92%, 24.25%, and 23.70%, respectively, highlighting the varied impact across different populations [12,14,15]. Additional studies reported even lower obesity prevalence rates of 13.77% and 14.60% [17,18]. When examining the odds ratios for high BMI, significant variations were observed. Bodicoat et al. reported an odds ratio of 0.04 (0.00-0.08), indicating a very low association, while Chowdhury et al. found a stronger association with an odds ratio of 1.44 (1.13-1.84) [11,19]. Increased waist circumference was also a notable factor, with three studies reporting odds ratios of 0.10 (−0.04, 0.25), 2.758 (1.238-6.265), and 2.03 (1.07-3.77) [17-19]. Abdominal obesity demonstrated a strong correlation with diabetes risk. Ton et al. reported an odds ratio of 1.50 (1.31-1.80), and Lu et al. found an odds ratio of 1.55 (1.08-2.24), underscoring the importance of abdominal fat as a predictor of diabetes [14,15]. Dyslipidemia was prevalent in the obese population, with Ton et al. observing a prevalence of 12.20% [15]. Elevated triglyceride levels were also a concern, with Shrestha et al. [12] and Mansour et al. [16] reporting odds ratios of 2.1 (1.8-2.6) and 0.65 (0.491-0.871), respectively, while another three studies reported odds ratios of 2.18 (1.40-3.38), 1.57 (1.32-1.87), and 2.2 (1.8-2.7), respectively, for hypertension [11-13]. Hypertension emerged as a significant comorbidity, with three studies reporting odds ratios of 2.18 (1.40-3.38), 1.57 (1.32-1.87), and 2.2 (1.8-2.7) [11-13]. Furthermore, Ton et al. documented a hypertension prevalence of 30.6% among their study participants, highlighting the high burden of hypertension in obese populations [15].

Various early intervention and prevention strategies were recommended by the authors of the included studies (Table 4), targeting the increasing prevalence of diabetes and obesity. Mostly, the authors indicated and emphasized the control of obesity to prevent diabetes, as Bodicoat et al. considered that as fast food consumption has been associated with an increased risk of type 2 diabetes and obesity, it has consequences for both public health initiatives to prevent diabetes and for those providing planning clearance for new fast-food establishments. Hence, public health measures could reasonably target fast-food establishments [19]. Similarly, Chowdhury et al. agreed that age-specific prevention efforts are required at the primary level, including advocacy campaigns about the negative effects of fast food habits and the futility of physical mobility, work, or exercise, and at the secondary level, like incorporating the issue text on the causes and consequences of diabetes and non-communicable diseases into the school-level curriculum [11].

**Table 3:** Screening and risk factors observed in the study population (OR 95% CI and %) aOR (95%CI): Adjusted Odds Ratio with 95% Confidence Interval; LDL: Low-Density Lipoprotein; HDL: High-Density Lipoprotein; HTN: Hypertension; BMI: Body Mass Index; NR: Not reported

Author	Obesity/High BMI (aOR (95%CI)	Abdominal obesity and Compound obesity (aOR (95%CI) (%)	Central obesity (aOR (95%CI) (%)	Dyslipidaemia (aOR (95%CI) (%)	Triglycerides (aOR (95%CI) (%)	LDL (aOR (95%CI) (%)	HDL (aOR (95%CI) (%)	HTN (aOR (95%CI) (%)	Waist circumference/waist to hip ratio
Chowdhury et al. 2022 [11]	1.44 (1.13 to 1.84)	-	-	-	-	-	-	1.5 (1.32-1.87)	
Shrestha et al. 2022 [12]	Overweight and obese: 2.0 (1.6 to 2.4)	-	-	-	2.1 (1.8-2.6)	-	-	2.2 (1.8-2.7)	
	14.60%	-	-	-	13.40%	-	-	-	
Al Thani et al. 2021 [13]		-	2.08 (1.02–4.26)	-	-			2.8 (1.40-3.38)	
	41.50%		66.20%	-	-	-	-	-	
Lu et al. 2021 [14]	Overweight and general: 0.04 (0.00- 0.08)	Abdominal: 1.55 (1.08–2.24)	-	-	-	-	-	-	
	24.25%	16.62%	-	-	-	-	-	-	
Ton et al. 2019 [15]	1.36 (1.16, 1.58)	Abdominal 1.5 (1.31-1.80)		1.45 (1.21, 1.73)	-	-	-		Waist- hip ratio: 1.65 (1.33, 2.05)
	23.70%	45.60%		12.20%	-	-	-	HTN:30.6%	Waist- hip ratio 80.9%;
Mansour et al. 2020 [16]	0.667 (0.485-0.918)	-	-	-	0.65 (0.491-0.871)	0.906 (0.742-1.105)	1.540 (1.098-2.161)		
	42.30%	-	-	-	43.40%	-	37.30%		
Aldossari et al. 2018 [17]	2.86 (0.98–8.37)	-	-	-	-	-	-		Waist circumference: 2.03 (1.07–3.77)
	13.77%	-	-	-	-	-	-		15.33%
Ranabhat et al. 2017 [18]	High BMI: 0.409 (0.069–2.422)	-	-	-	-	-	-	NR	Waist circumference: 2.758 (1.238–6.265)
	27.92%	-	-	-	-	-	-		67.53%
Bodicoat et al. 2015 [19]	High BMI: 0.04 (0.00- 0.08)	-	-	-		−0.01 (−0.01- −0.00)	0.00 (−0.00-0.00)	NR	Waist circumference: 0.10 (−0.04, 0.25);

**Table 4:** Early intervention and prevention strategies DM: Diabetes Mellitus; T2DM: Type 2 Diabetes Mellitus; DMT2: Diabetes Mellitus Type 2

Author	Early intervention/prevention strategy
Chowdhury et al. 2022 [11]	There is a need for a primary (awareness campaign about the adversity of fast food habits and the impotence of physical mobility/ work/exercise) and secondary (incorporate the issue text on the causes and consequences of diabetes and non-communicable diseases in the school-level curriculum) prevention efforts tailored to age-specific populations.
Shrestha et al. 2022 [12]	Targeted DM prevention and control interventions, especially to those population groups with higher chances of DM occurrence, in addition to prevention and control of the biological risk factors associated with DM through appropriate measures, would help curb the prevalence of diabetes.
Al-Thani et al. 2021 [13]	Lifestyle interventions targeting people living with pre-DM should also be a national priority to halt future progression to DM. Population-based prevention strategies addressing these conditions simultaneously are essential to reduce the growing disease burden of these conditions and their associated disease sequelae.
Lu et al. 2021 [14]	More attention should be paid to preventing obesity among people younger than 60 years, and increased control of cigarette or alcohol abuse should also be considered. With the increasingly high burden of diabetes in China, obesity management should be established to prevent or delay the development of T2DM.
Ton et al. 2019 [15]	There is a need for the government and professional bodies to implement public health interventions targeting middle-aged Vietnamese adults to prevent and control prediabetes and diabetes nationwide.
Mansour et al. 2020 [16]	Future research should be done, in prospective longitudinal studies, to explore risk factors and their association or causation effects on the prevalence of DMT2 in smaller cities and how this reflects on the wider community. A comprehensive approach is needed to promote healthy lifestyles and avoid the burden of urbanization’s negative health behaviours in communities of this size.
Aldossari et al. 2018 [17]	Before the ongoing transmission of diabetes reaches an epidemic threshold, the task force for the prevention, early diagnosis, prompt treatment, protection against its complications, and health education should be activated.
Ranabhat et al. 2017 [18]	The future health programs should focus on generating awareness on the health effects of sedentary occupation, symptoms, and prevention of diabetes especially focused on people from lower socioeconomic groups. Increasing activity at the working conditions and measures for obesity control should be the priority of the non-communicable disease control program.
Bodicoat et al. 2015 [19]	Fast food outlets have implications for diabetes prevention at a public health level and for those granting planning permission to new fast-food outlets as it is associated with increased risk of type 2 diabetes and obesity. Fast food outlets might be a reasonable target for public health interventions.

While Ranabhat et al. were of the opinion that future health initiatives should concentrate on raising public knowledge of the adverse health consequences of sedentary work, diabetes symptoms, and diabetes prevention, with a particular emphasis on those from lower socioeconomic backgrounds, the non-communicable disease control program should prioritize improving working conditions and taking steps to control obesity [18]. Aldossari et al. also commented that the task force for diabetes prevention, early diagnosis, prompt treatment, prevention against complications, and health education should be established before the continued spread of the disease reaches an epidemic threshold [17]. Ton et al. suggested that the government and professional associations must concentrate on public health initiatives to prevent the increasing prevalence of diabetes and obesity [15]. Mansour et al. recommended that prospective longitudinal studies should be used in the future to investigate risk variables and their relationship to or causal impact on the prevalence of type 2 diabetes, as well as the implications for the population. A comprehensive strategy is required to encourage healthy lifestyles and prevent the poor health behaviors associated with urbanization [16].

Furthermore, Al-Thani et al. added that to prevent future development of diabetes, lifestyle interventions for individuals with pre-diabetes should also be a priority. To mitigate the increasing burden of these disorders and the related health consequences, population-based preventive measures are crucial [13]. Lu et al. recommended that prevention of obesity in individuals under 60 years of age should receive more focus, and measures to curb alcohol or cigarette consumption should also be taken into account [14]. Shrestha et al. also agreed that the prevalence of diabetes could be reduced by targeted diabetes prevention and control treatments, particularly for demographic groups with higher odds of diabetes incidence, as well as effective strategies for the prevention and management of the biological risk factors associated with diabetes [12]. Overall, the results suggest that obesity is an independent risk factor for the development of diabetes among obese individuals, and interventions like increasing awareness of healthier lifestyles are needed to manage the rising burden of these diseases.

Discussion

This study aimed to assess various risk factors that may contribute to the incidence of diabetes among the obese population, while additionally evaluating different preventive strategies that can promote early intervention for the prevention of diabetes in this population. Findings suggest that obesity alone is a significant contributor to the occurrence or onset of diabetes, while the presence of other risk factors, including hypertension, dyslipidemia, elevated triglycerides, or HDL and LDL levels, may further increase the risk of diabetes and its associated complications among this population. The diverse prevalence rates and odds ratios across studies illustrate the complex and multifaceted nature of these health issues. Preventive strategies emphasize early intervention through increasing awareness and educating communities about risk factors and lifestyle interventions, including the limitations of fast food diets for the prevention of diabetes and weight control.

Mechanistic Insights

Throughout the world, adults and children are affected by obesity, a chronic metabolic condition. Since obesity is known to be the primary risk factor for many non-communicable diseases, including type 2 diabetes, it has emerged as one of the leading causes of mortality. Because of their close relationship, the term "diabesity" has emerged to refer to the condition in which most people with diabetes are overweight or obese [20]. One of the primary mechanisms linking obesity to diabetes is insulin resistance. In obesity, excess fat accumulates in adipose tissue, leading to an increase in free fatty acids (FFAs) in the bloodstream. Elevated FFAs interfere with insulin signaling pathways, causing insulin resistance in peripheral tissues such as muscle and liver [21]. Additionally, obesity alters the secretion of adipokines (e.g., leptin, adiponectin) and increases the production of pro-inflammatory cytokines (e.g., TNF-α, IL-6) from adipose tissue, promoting insulin resistance and chronic inflammation, which further impairs glucose metabolism [22]. Beta-cell dysfunction is another critical factor in the relationship between obesity and diabetes. Chronic exposure to high levels of FFAs and glucose can lead to lipotoxicity and glucotoxicity, respectively. These conditions damage pancreatic beta cells, reducing their ability to produce and secrete insulin. Moreover, obesity-induced endoplasmic reticulum (ER) stress in beta cells disrupts normal protein folding and processing, contributing to beta-cell apoptosis and dysfunction [23].

Baum et al. presented the initial research on the association between weight gain in infancy and subsequent development of type 1 diabetes in 1975. In line with the study by Baum et al., there may be an association between hormonal dysregulation and overfeeding. While one of the most widely recognized ideas elucidating the relationship between body mass and type 1 diabetes is Wilkin's accelerator hypothesis as stated by the authors of this notion, type 1 diabetes risk increases with increased body weight in younger age groups. Age at diagnosis and BMI have an inverse relationship. Furthermore, diabetes can be identified sooner in young children as they gain more weight. This can be explained by the fact that those who are genetically inclined to diabetes develop type 1 diabetes sooner since weight gain increases insulin resistance [24,25].

While insulin resistance is linked to obesity and type 2 diabetes, even though they are insulin resistant, the majority of obese individuals do not experience hyperglycemia. The islet of Langerhans' pancreatic β-cells produces enough insulin in sufficient quantities to counteract declines in insulin levels under typical conditions, preserving normal glucose tolerance [26]. Endothelial dysfunction and obesity/insulin resistance are associated with diabetes and prediabetes conditions throughout the natural history of type 2 diabetes; this includes patients with impaired glucose tolerance and/or impaired fasting glucose. Type 2 diabetes is a result of insulin resistance and obesity, which can be fully compensated for by β-cells in the absence of sufficient insulin sensitivity. Since obese individuals release nonesterified fatty acids from their adipose tissue, β-cell malfunction and insulin resistance may be related [27].

Altered gut microbiota also plays a significant role in this relationship. Obesity is associated with changes in gut microbiota composition, known as dysbiosis. Dysbiosis can affect metabolic processes and promote inflammation, influencing insulin sensitivity and glucose homeostasis. The gut microbiota produces short-chain fatty acids (SCFAs), which play a role in energy metabolism. Alterations in SCFA production due to obesity can impact insulin sensitivity and glucose regulation [28]. Hormonal changes are another contributing factor. Leptin, a hormone produced by adipose tissue, regulates appetite and energy balance. In obesity, leptin resistance develops, impairing its ability to suppress appetite and promote energy expenditure, contributing to weight gain and insulin resistance. Additionally, hormones such as GLP-1, which are secreted by the gut in response to food intake, enhance insulin secretion. Obesity can impair incretin responses, reducing insulin secretion and glucose tolerance. Genetic and epigenetic factors also link obesity and diabetes. Certain genetic variants increase susceptibility to both obesity and diabetes, affecting insulin signaling, adipocyte differentiation, and other metabolic processes. Furthermore, environmental factors such as diet and physical activity can lead to epigenetic changes (e.g., DNA methylation, histone modification) that influence gene expression and metabolic pathways, connecting obesity to diabetes [29]. Lastly, energy imbalance and lifestyle factors are pivotal. High-calorie diets and sedentary lifestyles contribute to energy imbalance, weight gain, and obesity. These lifestyle factors exacerbate insulin resistance and beta-cell dysfunction, increasing the risk of diabetes [30].

Risk Factors and Clinical Implications

The findings of our study demonstrate that in addition to obesity, which itself is a strong predictor of diabetes, among the study participants, other risk factors identified were dyslipidemia, elevated HDL and LDL levels, and hypertension. The incidence of these risk factors among obese individuals may incline them more towards the development of complications and affect their quality of life. Similarly, Niswender et al. further explained that an atherogenic lipid profile, type 2 diabetes, obesity, and overweight are closely related and considerably increase the risk of cardiovascular morbidity and mortality. Even though there is evidence that losing weight has major advantages, individuals with type 2 diabetes frequently report that lifestyle modifications are insufficient to achieve or sustain a goal BMI of less than 25 kg/m^2^, HbA1c level of less than 7%, and blood pressure and cholesterol targets. Most people gain weight instead of losing it, especially when their glycaemic management is intensified [31]. Additionally, results of a study by Moosaie et al. showed that obesity was found to be independently linked to cardiovascular diseases, neuropathy, nephropathy, and retinopathy in individuals with Type 1 and Type 2 diabetes, as well as retinopathy exclusively in Type 1 diabetes. For Type 1 and Type 2 diabetes, respectively, there was a stronger correlation between obesity, retinopathy, and neuropathy [32]. Another cohort study by Regmi et al. concluded that the development of type 2 diabetes was independently predicted by age (≥44 years), obesity in women, and pre-diabetes in males [33]. Likewise, Sanada et al. also commented that in patients who are overweight or obese, high BMI is a dose-dependent and independent risk factor for type 2 diabetes [34].

Adding further to this context, Jatoi et al. also revealed that almost 89.6% of type 2 diabetes patients suffer from obesity and are overweight, indicating it is a significant predictor of diabetes [35]. Results of a study by Yamada et al. revealed that diabetes, hypertension, and dyslipidemia with and without hyper-low-density lipoprotein-cholesterolemia were found in 9.7%, 41.0%, 63.8%, and 19.5% of the population, respectively, and these disorders were more common as obesity increased. At a BMI of ≥ 35 kg/m2, compared with normal weight, the risk for diabetes and hypertension was markedly increased (odds ratios of 12.95 and 19.44, respectively), and the risk for dyslipidemia with and without hyper-low-density lipoprotein-cholesterolemia was modestly increased (odds ratios of 2.59 and 3.65, respectively) [36].

Moreover, findings of a community-based study indicated that factors including hypertension (OR=1.768, P=0.042), hyperuricemia (OR=2.263, P=0.003), disease course (OR=1.050, P=0.007), glycosylated haemoglobin A1c (OR=1.358, P<0.001), systolic blood pressure (OR=1.027, P<0.001), and hyperuricemia (OR=2.263, P=0.003) were associated with an increased risk of developing diabetic nephropathy among obese individuals [37]. Similarly, in our findings, almost three studies reported significant odds ratios for hypertension, making it a strong predictor and risk factor for the development of diabetes in obese individuals, while a few studies also reported significant odds ratios for elevated HDL and LDL levels. Our findings are inconsistent with the findings of existing literature, hence indicating that obesity itself is an independent predictor of the development of diabetes among obese individuals; therefore, preventive strategies should be developed and tailored accordingly, focusing on weight loss and control recommendations.

Recommendations and Early Intervention Strategies

According to statistics, almost 20%-30% of patients with diabetes usually experience issues before receiving a diagnosis, and approximately half of people with diabetes go undiagnosed. Therefore, there is an urgent need for an alternate screening method to enable an earlier diagnosis of diabetes. More useful and practical risk assessment tools than traditional blood glucose screening tests have been developed for the prediction of type 2 diabetes. These tools are based on self-assessed biochemical measures or genetic markers, and they allow interventions to be given to individuals with impaired glucose tolerance to postpone the onset of type 2 diabetes [6]. The first step in treatment is screening for diabetes and obesity, which frequently identifies people who are at risk but may not yet have obvious diseases. The United States Preventive Services Task Force advises screening for obesity in all adult populations. Excess adipose tissue is an indication of obesity. Calculating the BMI is the screening technique that is most frequently employed. Weight in kilograms divided by height in meters squared yields the BMI. When height and weight are entered into most electronic medical records, the BMI is automatically calculated. BMI can also be calculated using a variety of smartphone apps and internet calculators. The waist-to-hip ratio and waist circumference are two additional screening instruments. A waist circumference greater than 35 inches or a waist-to-hip ratio greater than 0.7 in females is indicative of increased visceral fat and risk of disease [38].

As an initial target for treatment, it is recommended to lose 5%-10% of baseline body weight. This amount of weight loss is linked to several health advantages, including a 0.6-1.0% decrease in Haemoglobin A1C. But even a 2-5% weight loss results in a clinically significant drop in fasting blood glucose (20 mg/dL). For individuals with prediabetes, the American Diabetes Association recommends a 7% body weight loss to help prevent the onset of type 2 diabetes [39]. Cohort studies including adult patients with type 2 diabetes have demonstrated that a 25% decrease in all-cause mortality was observed in those who dropped 9-13 kg in comparison to patients who were weight-neutral [40]. The findings of this review suggest increasing awareness regarding the beneficial effects of weight loss on the prevention of diabetes and associated complications among obese individuals. Additionally, fast-food consumption should be discouraged and limited to facilitate healthy lifestyle choices. Moreover, special emphasis should be laid on health promotion and education in this cohort so the burden of these diseases can be reduced.

Similarly, Gruss et al. recommended maintaining an emphasis on the objectives of fundamental lifestyle change program trials and their global applications, as well as to target the most vulnerable populations through both in-person and virtual program delivery methods. Efforts targeting high-risk populations must be supplemented by whole-population strategies such as socioeconomic policies, healthy food promotion, environmental/systems modifications, and increasing awareness [41]. Additionally, families at risk for type 2 diabetes benefit from receiving diabetes education, which is well-received and has a positive impact on pertinent risk variables [42]. To avert the escalating burden of the disease, effective steps to reduce diabetes incidence and increase awareness should be adopted [43]. Balagopal et al. also described that a successful educational intervention improved the food habits of those with pre-diabetes and diabetes and reduced some obesity parameters [44]. Achieving a healthy body weight by combining dietary changes with physical activity should be the goal of diabetes prevention education. Behavioral therapy, including goal setting, self-monitoring, and motivational interviewing, can enhance patient engagement and adherence [45]. Regular follow-ups, support groups, and the use of technology can provide ongoing support and motivation [46]. Attempting to dispel long-held myths and replace them with factual information derived from science should be the first step in any educational programme. For the campaign to be effective, it has to be customized to the requirements and culture of that region. Establishing environments that support a healthy way of living is crucial [47].

Franz highlighted that studies have shown the effectiveness of nutrition therapy in managing diabetes at all stages, from obesity and prediabetes to diabetes itself. Achieving modest weight loss is particularly important for those with prediabetes or who are overweight or obese. For type 2 diabetes, the key goals of dietary therapy include improving blood pressure, lipid levels, and blood sugar control. Reducing caloric intake is crucial for meeting these goals. While lowering energy consumption can lead to weight loss in some individuals, it can also help maintain weight loss or prevent further weight gain in others. Throughout this process, weight loss medications and metabolic surgery have proven to be effective treatment options [48].

Secondary prevention aims to halt the progression of obesity and diabetes, thereby reducing associated morbidity and mortality. Clinical inertia, the failure to initiate or intensify therapy when indicated, is a significant barrier to effective diabetes management. Strategies to reduce clinical inertia include ensuring healthcare providers are well-versed in current clinical guidelines, implementing electronic health records with clinical decision support systems, and employing a team-based approach involving endocrinologists, dietitians, nurses, and pharmacists [49].

When lifestyle interventions alone are insufficient to achieve glycemic and weight targets in patients with obesity and diabetes, the early use of combination therapy with highly tolerated doses of medications becomes crucial. Combination therapy, which involves using two or more medications with complementary mechanisms of action, can significantly enhance treatment efficacy, improve patient outcomes, and reduce the risk of complications associated with these conditions. This approach addresses the multifactorial nature of diabetes and obesity, targets multiple pathophysiological pathways simultaneously, and prevents disease progression [50]. Commonly used medications in combination therapy include metformin, glucagon-like peptide-1 (GLP-1) receptor agonists, sodium/glucose cotransporter 2 (SGLT2) inhibitors, dipeptidyl peptidase-4 (DPP-4) inhibitors, and thiazolidinediones (TZDs). Metformin, often the first-line treatment, reduces hepatic glucose production and improves insulin sensitivity, while GLP-1 receptor agonists like liraglutide and semaglutide enhance glucose-dependent insulin secretion, suppress glucagon secretion, and promote weight loss. SGLT2 inhibitors, such as empagliflozin and canagliflozin, increase urinary glucose excretion, thereby lowering blood glucose levels and promoting weight loss [51]. DPP-4 inhibitors enhance incretin hormone activity, increasing insulin secretion and decreasing glucagon levels. TZDs improve insulin sensitivity in peripheral tissues and can be beneficial in specific patient populations despite their association with weight gain [25,52-54]. Studies have demonstrated that early use of combination therapy can lead to better long-term glycemic control compared to sequential monotherapy. For example, the VERIFY study showed that early combination therapy with metformin and vildagliptin resulted in sustained glycemic control and delayed treatment failure over a five-year period compared to metformin alone [55]. This approach also addresses various aspects of metabolic syndrome, which often accompanies obesity and diabetes, reducing insulin resistance, improving beta-cell function, and lowering cardiovascular risk factors more effectively than monotherapy [50]. When initiating combination therapy, it is essential to consider the patient's overall health status, potential side effects, and preferences. Tolerability and adherence are critical factors in the success of combination therapy. Medications should be chosen based on their complementary mechanisms, efficacy, safety profiles, and impact on weight and cardiovascular health. 

A comprehensive and patient-centered approach is essential to prevent the worsening burden of diabetes and obesity, enhance patient well-being, and reduce morbidity and mortality. This approach involves regular monitoring and adjustment of therapy, educating patients on the importance of medication adherence and lifestyle modifications, and addressing mental health issues such as depression and anxiety [56]. 

Strengths, limitations, and future research directions

Our review presents findings from the latest evidence, including studies from the past 10 years and high-quality research, which are key strengths of our review. Moreover, our systematic search methodology and comprehensive analysis of relevant keywords further enhance the rigor and robustness of this study. Further, this systematic review addresses key gaps in the understanding and management of diabetes among obese populations by highlighting the complex interplay between obesity and other significant risk factors such as hypertension, dyslipidemia, and elevated triglycerides. It reveals substantial variation in obesity prevalence and associated risk factors across different populations, underscoring the need for tailored interventions that account for specific local contexts. The review identifies a lack of standardized criteria in assessing diabetes risk factors, pointing to the necessity of uniform protocols for more reliable research outcomes. Additionally, it emphasizes the importance of early intervention and preventive strategies specifically designed for the obese population, including lifestyle changes, nutrition therapy, and the use of weight loss medications and metabolic surgery. Finally, the review underscores the need for educational and awareness campaigns to promote healthy lifestyle choices and early screening for diabetes and obesity.

Our study also has certain limitations; heterogeneity among the reporting of risk factors in the studies was observed, as all of the included studies did not report on all risk factors, which may limit the generalizability of our findings. Additionally, variations among the intrinsic characteristics of studies may further add to heterogeneity. Moreover, considering only English papers may have resulted in overlooking some significant studies, which further adds to the limitation of our study. Since the burden of both obesity and diabetes is on the rise, action for prevention is a dire need. Screening for obese individuals shall be held regularly in addition to health promotion and education activities specifically targeting weight loss and awareness programs at the community level for the prevention of diabetes and associated risk factors. Research studies in the future should target evaluating the efficacy of various awareness campaigns so uniformly targeted guidelines are available for implementation in clinical practice that not only educate patients and raise awareness but also motivate them for screening for risk factors and adaptation of healthy lifestyles so diabetes and obesity are both managed effectively. Furthermore, comprehensive systematic reviews and meta-analyses are also needed in the future to present more evidence-based findings among different age-specified populations so a better understanding of the risk factors can be achieved and addressed.

## Conclusions

Globally, diabetes and its ramifications place a significant health burden on society. Since obesity is a major independent risk factor for the development of diabetes, addressing and managing obesity is critically important clinically. This review provides an in-depth analysis of the relationship between obesity and diabetes, emphasizing context-specific, standardized, and early intervention strategies to better manage and prevent diabetes in diverse populations. Targeted early interventions, such as screening for risk factors, health promotion, and educational activities, can facilitate the adoption of healthier lifestyles and significantly reduce the burden of these diseases. Furthermore, future research can enhance clinical practice by developing effective, standardized preventive guidelines aimed at reducing the likelihood of diabetes among obese individuals and promoting weight loss.
